# Complete Genome Sequences for Two *Talaromyces marneffei* Clinical Isolates from Northern and Southern Vietnam

**DOI:** 10.1128/MRA.01367-19

**Published:** 2020-01-09

**Authors:** Christina A. Cuomo, Terrance Shea, Thu Nguyen, Philip Ashton, John Perfect, Thuy Le

**Affiliations:** a Broad Institute of MIT and Harvard, Cambridge, Massachusetts, USA; b Division of Infectious Diseases, Duke University School of Medicine, Durham, North Carolina, USA; c Oxford University Clinical Research Unit, Ho Chi Minh City, Vietnam; Vanderbilt University

## Abstract

Talaromyces marneffei is a thermally dimorphic fungus endemic in China and Southeast Asia that causes fatal infections in immunocompromised individuals, particularly in patients with advanced HIV disease. Here, we report the complete genome sequences of two clinical isolates from northern and southern Vietnam.

## ANNOUNCEMENT

The thermally dimorphic fungus Talaromyces (formerly Penicillium) *marneffei* causes fatal infections in immunocompromised individuals. In Vietnam, Thailand, and southern China, where *T. marneffei* is highly endemic, talaromycosis is a leading opportunistic infection and cause of death in HIV-infected individuals. The on-treatment mortality rates in HIV-infected and non-HIV-infected individuals approach 30% and 50%, respectively (1–3).

Two isolates of T. marneffei were collected from patients enrolled in an antifungal clinical trial in Vietnam (4). As prior multilocus sequence typing (MLST) analysis suggested a geographic substructure of *T. marneffei* in Southeast Asia (5), we selected one isolate from northern Vietnam (11CN-20-091) and one isolate from southern Vietnam (11CN-03-130) for genome sequencing. The isolates were cultured in Sabouraud dextrose agar (SDA) in the yeast form at 37°C for 5 days. DNA was prepared using the MasterPure yeast DNA purification kit (Epicentre). Oxford Nanopore libraries were constructed using the 1D ligation kit (catalog number SQK-LSK109) and loaded on a FLO-MIN106D flow cell for each sample on a GridIon instrument. Base calling was performed using Albacore v2.3.4 for 11CN-20-091 and using MinKNOW v3.1.20 for 11CN-03-130. A total coverage of 170× was generated for 11CN-03-130, and 152× coverage was generated for 11CN-20-091 (Table 1). The reads for each sample were assembled using Canu v1.5 (6), with the parameters “genomeSize=29000000” and “correctedErrorRate=0.075.” Next, the nanopore reads were aligned to the Canu assembly with minimap2 v2.9r720 (7), with the parameter “-ax map-ont,” and each assembly was polished with Nanopolish v0.11.0.

**TABLE 1:** *Talaromyces* genome statistics

Genome statistic	Data for *Talaromyces* isolate:	
	11CN-03-130	11CN-20-091
No. of Nanopore reads	1,331,235	943,888
Nanopore coverage (×)	175	157
No. of Illumina reads (Flex library)	5,009,866	3,604,362
No. of Illumina reads (Nextera library)	13,936,480	18,321,844
Illumina coverage (both libraries) (×)	110	82
No. of contigs	9	8
Maximum contig length (bp)	6,376,262	6,464,005
Contig *N*_50_ (bp)	3,743,714	3,704,010
Total contig length (bp)	28,216,733	28,198,338
Assembly GC content (%)	46.79	46.76
No. of protein-coding genes	10,025	9,994
BUSCO (%)	97.7	97.40

Two sets of Illumina data were used for error correction. One library for each sample was constructed using the DNA Flex Illumina protocol and sequenced on an iSeq instrument to generate paired 250-base reads (Table 1). A second library for each sample was constructed by Macrogen using the Nextera kit and sequenced on a HiSeq 4000 instrument to generate paired 101-base reads (Table 1). The assemblies were polished with all Illumina data using three rounds of alignment with BWA-MEM v0.7.7-r411 (8) and Pilon v1.23 (9) correction. Alignment of the assembly of 11CN-03-130 to that of 11CN-20-091 using Nucmer v3.1 (10) identified candidate rearrangement events. Nanopore and Illumina read alignments were visually inspected across these junctions in IGV v2.1.5 (11); where this revealed misassemblies, contigs were manually broken, correctly joined, and polished as described above. Contig alignments suggested that two contig joins could be made in 11CN-03-130, and one join could be made in 11CN-20-091; all junctions were validated and polished as described above. One intrachromosomal rearrangement supported by read alignments was identified between these two isolates.

The assembly of 11CN-03-130 consists of nine contigs which have an *N*_50_ value of 3.74 Mb and a total length of 28.22 Mb. Of the 8 largest contigs, 7 have telomeric repeats (TTAGG[GA]) at both ends; contig000003 has telomeric repeats at the end, and the start consists of rRNA gene repeats. The smallest contig (82.9 kb) consists of ∼11 tandem copies of rRNA gene repeat units and is likely linked to the start of contig000003. The assembly of 11CN-20-091 consists of 8 contigs that have telomeric repeats at both ends, an *N*_50_ value of 3.70 Mb, and a total length of 28.20 Mb. The GC content of both assemblies is 46.8%. The total size is similar to those previously noted for draft assemblies of *T. marneffei* isolates ATCC 18225 (28.6 Mb) (12) and PM1 (28.9 Mb) (13).

Gene structures were predicted using transcriptome sequencing (RNA-seq) data from yeast and mycelia (14). RNA-seq reads were aligned to each assembly using STAR v2.7 (15), with the parameter “-alignIntronMax 10000,” and alignments were input to BRAKER v1.7 (16). A total of 10,025 genes were predicted for 11CN-03-130, and 9,994 genes were predicted for 11CN-20-091. BUSCO v3 (17) identified 97.7% and 97.4% of the pezizomycotina_odb9 gene set in the 11CN-03-130 and 11CN-20-091 gene sets, respectively.

### Data availability.

The sequence, assembly, and annotation reported here are available in GenBank under BioProject accession number PRJNA522919. Raw sequence reads have been deposited in the NCBI Sequence Read Archive for 11CN-03-130 (Oxford Nanopore data, accession number SRR8592562; Illumina iSeq data, accession number SRR8784960; and Illumina HiSeq 4000 data, accession number SRR10359552) and for 11CN-20-091 (Oxford Nanopore data, accession number SRR8592561; Illumina iSeq data, accession number SRR8784959; and Illumina HiSeq 4000 data, accession number SRR10359551). Annotated assemblies are deposited under GenBank accession number WINJ00000000 for 11CN-03-130 and under accession numbers CP045653 to CP045660 for 11CN-20-091.
